# Autophagy and its role in osteosarcoma

**DOI:** 10.1002/cam4.5407

**Published:** 2023-02-15

**Authors:** Biao Ning, Yixin Liu, Tianhe Huang, Yongchang Wei

**Affiliations:** ^1^ Department of Radiation and Medical Oncology Zhongnan Hospital of Wuhan University Wuhan Hubei China; ^2^ Hubei Key Laboratory of Tumor Biological Behaviors Zhongnan Hospital of Wuhan University Wuhan Hubei China; ^3^ Hubei Cancer Clinical Study Center Zhongnan Hospital of Wuhan University Wuhan Hubei China

**Keywords:** autophagy, chemotherapy, osteosarcoma, progression, radiotherapy, immunotherapy

## Abstract

Osteosarcoma (OS) is the most common bone malignancy and preferably occurs in children and adolescents. Despite significant advances in surgery and chemotherapy for OS over the past few years, overall survival rates of OS have reached a bottleneck. Thus, extensive researches aimed at developing new therapeutic targets for OS are urgently needed. Autophagy, a conserved process which allows cells to recycle altered or unused organelles and cellular components, has been proven to play a critical role in multiple biological processes in OS. In this article, we summarized the association between autophagy and proliferation, metastasis, chemotherapy, radiotherapy, and immunotherapy of OS, revealing that autophagy‐related genes and pathways could serve as potential targets for OS therapy.

## Graphical abstract

In this review, we summarized the role of autophagy in OS proliferation, metastasis, chemotherapy, radiotherapy, and immunotherapy. And we think that autophagy‐related genes and pathways could serve as potential targets for OS therapy.

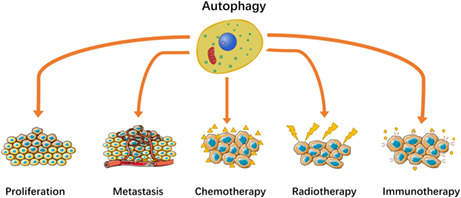

## 1 INTRODUCTION

Osteosarcoma (OS) is a highly aggressive bone malignancy which most commonly occurs in children and adolescents.^1^ There are approximately 4.4 cases of OS per million children reported annually.^2^, ^3^ OS tends to occur in the metaphysis of long bones, and the three sites with the highest incidence are distal femur, proximal tibia, and proximal humerus.^4^ Due to its high malignancy, early metastasis, and easily drug resistance, it is characterized by a high rate of morbidity and mortality as well as an extremely poor prognosis.^5^ Its current standard therapy is based on neoadjuvant chemotherapy (high‐dose methotrexate [MTX], doxorubicin [DOX], ifosfamide [IFO], and cisplatin [CIS]) followed by radical tumor resection surgery.^6^ In addition, radiotherapy and immunotherapy play an increasingly important role in the comprehensive treatment of OS.^7^ Nevertheless, 5‐year overall survival and treatment outcomes OS patients have remained largely unchanged over the past few decades, and prognosis remains poor for patients who develop metastases and/or relapse after treatment.^8^ Hence, there is an urgent demand to explore novel therapeutic targets to develop new therapeutic strategies in OS.

Autophagy is a kind of catabolic pathway which is strictly regulated and stress‐induced among eukaryotic cells. This process is manifested by the formation of double‐membrane vesicles (called autophagosomes) to engulf damaged organelles, unfolded proteins, pathogens, etc., followed by transporting them to lysosomes for breakdown and digestion.^9^ Autophagy is an important regulator of cellular homeostasis, and its dysfunction is related to multiple human diseases, including Alzheimer's disease, Parkinson's disease, and malignancy.^10^, ^11^ The role of autophagy in malignancy has become new focus of attention, and researches in this area have progressed significantly over the past few years.^12^, ^13^, ^14^, ^15^, ^16^ The function of autophagy in malignancy is complex and only partially understood, thus delaying the development of drugs targeting autophagy to treat cancer. The reported role of autophagy in malignancy performs like a double‐edged sword, which can not only inhibit tumorigenesis and development by reducing the accumulation of damaged proteins and organelles, but also act as a cell protective factor to promote tumor cell growth and metastasis.^17^, ^18^ For example, in precancerous lesions, many studies have shown that inducers of autophagy could prevent cancer progression.^13^ Conversely, in advanced cancers, both enhancement and inhibition of autophagy have been proposed as therapeutic targets.^12^, ^19^, ^20^ More importantly, more and more studies have confirmed that autophagy promotes resistance during cancer treatment.^21^, ^22^

**FIGURE 1:**
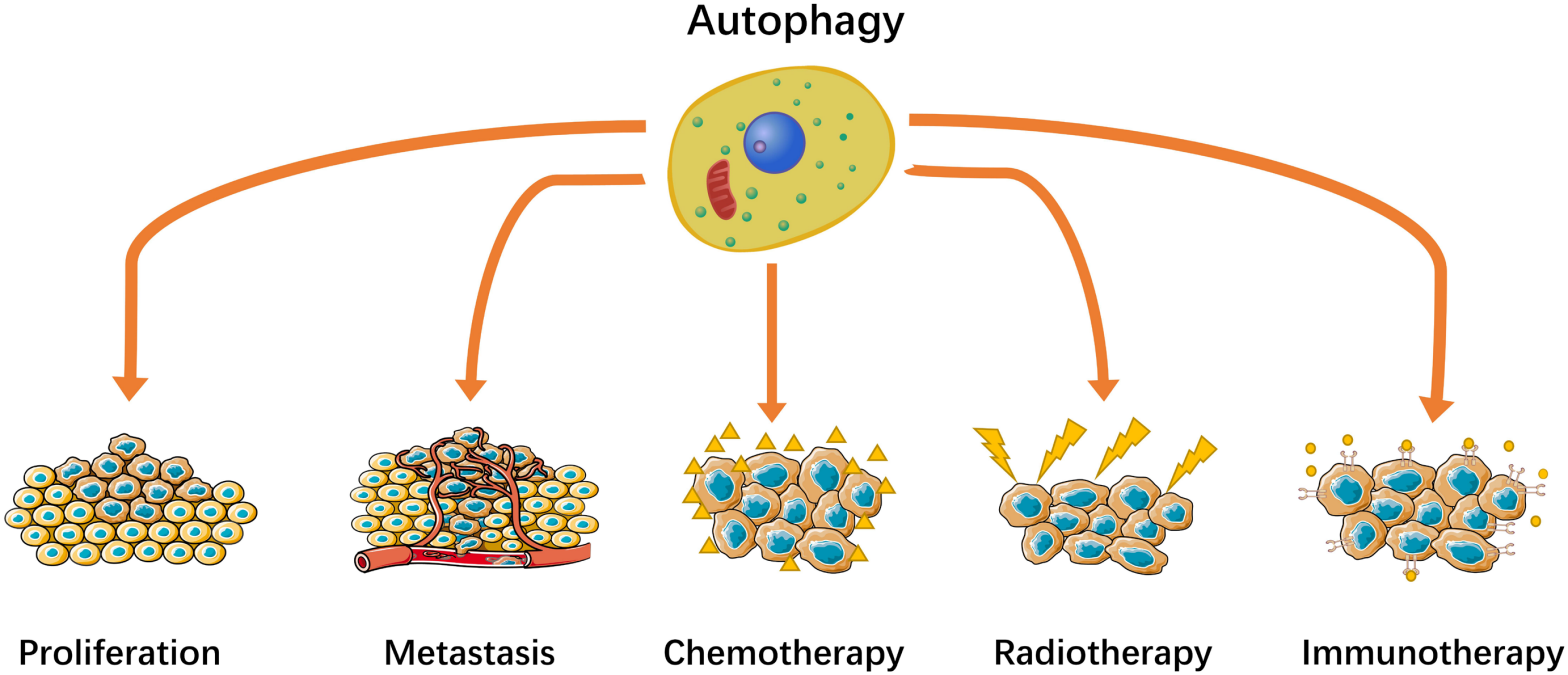
Autophagy involved in OS progression and treatment.

A dual role of autophagy is observed in multiple tumor types, as well as in osteosarcoma. On the one hand, OS tumor cells utilize autophagy as a means of therapy resistance, proliferation, and cancer stem cell protection.^23^ On the other hand, autophagy inhibits the proliferation and metastasis of osteosarcoma through inducing autophagic cell death, a physiological cell death which is considered to be type II programmed cell death.^24^ In this article, we summarized the association between autophagy and proliferation, metastasis, chemotherapy, radiotherapy, and immunotherapy of OS, revealing that autophagy‐related genes and pathways could serve as potential targets for OS therapy (Figure 1).

## 2 AN OVERVIEW OF AUTOPHAGY

### 2.1 Autophagy classification

Autophagy can be divided into three subtypes: chaperone‐mediated autophagy (CMA), microautophagy, and macroautophagy.^25^, ^26^, ^27^ CMA, which occurs primarily in mammalian cells, is a highly specific process. Proteins are firstly recognized by heat shock cognate protein 70 (HSC70), and then, through the action of the integral membrane receptor lysosome‐associated membrane protein‐2A (LAMP‐2A), internalized into lysosome for degradation.^28^ Microautophagy is the direct phagocytosis of cellular components through invagination of the late endosome followed by the transportation of cellular components into lysosomes for degradation by lysosomal hydrolases, a process that does not involve autophagosome formation.^29^ Macroautophagy is the process by which cells form double membrane autophagic vesicles (AVs). After AVs phagocytose cellular components, they mature into autophagosomes, which fuse with lysosomes. Hydrolases located in lysosomes degrade cellular components into smaller, usable molecules such as fatty acids, amino acids, sugars, nucleotides, etc. for recycling and reentry into biosynthesis.^30^ Macroautophagy, the most prevalent subtype and extensively studied form of autophagy, will be the focus of this review (hereafter simply referred to as autophagy).

### 2.2 The processes and molecular mechanisms of autophagy

**FIGURE 2:**
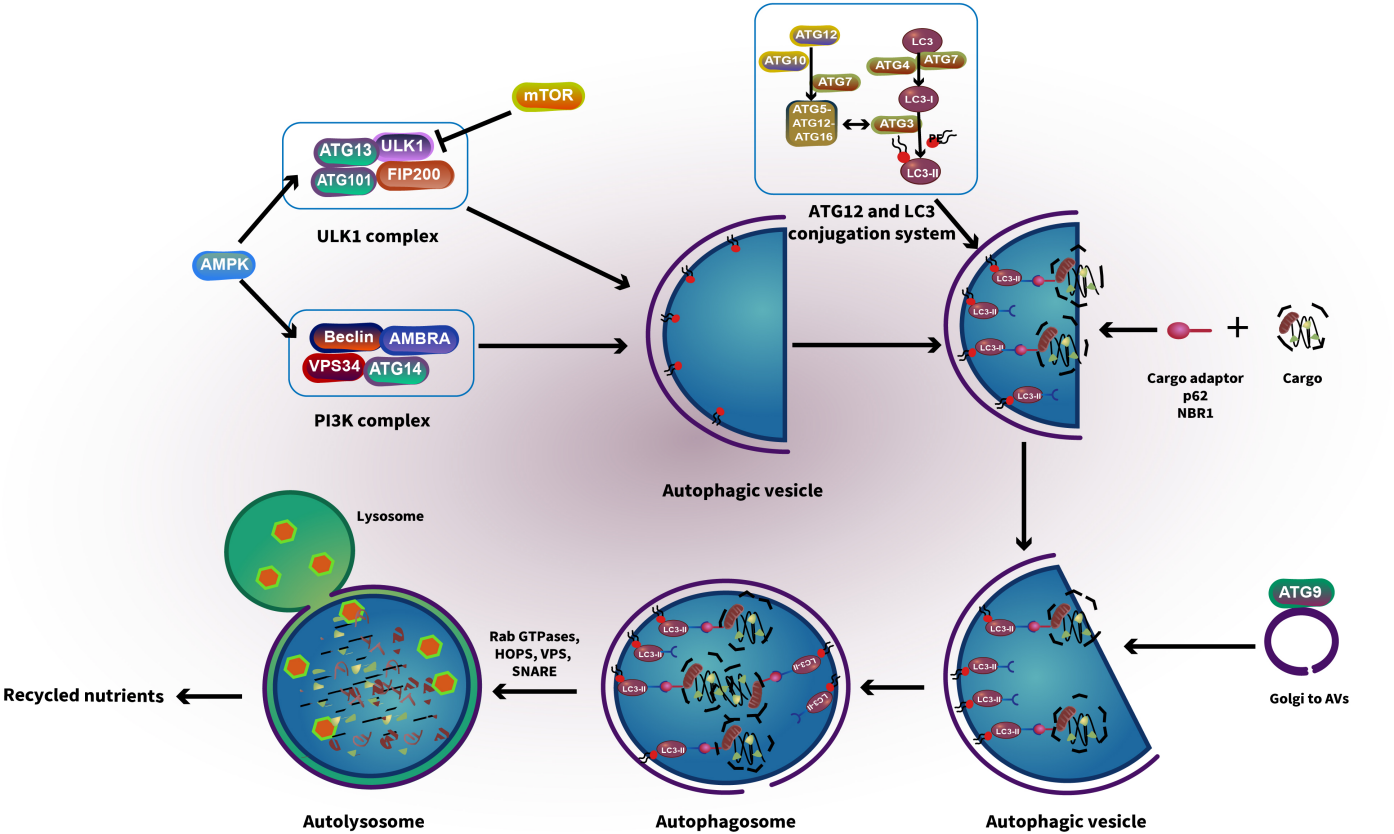
The main processes of autophagy and their molecular mechanisms.

The process of autophagy involves more than 30 autophagy‐related genes (ATGs) and includes the following seven consecutive steps (Figure 2). And the ATG genes regulate steps 1 to 5, while genes common to other endosome/lysosomal pathways promote steps 6 and 7.^31^

1. Under situations of nutrient deprivation, hypoxic conditions, and/or chemotherapeutic treatment, mTORC1 is inhibited or AMPK is activated, which then activates the downstream serine/threonine Unc‐51‐like kinase 1 (ULK1). The activated ULK1 can interact with both the focal adhesion kinase family interacting protein of 200 kDa (FIP200) and the conjugate of autophagy‐related protein 13 (ATG13) and ATG101 to form the ULK1‐FIP200‐ATG13‐ATG101 complex, which is also known as the ULK1 initiation complex.^32^, ^33^
2. Upon its formation, the ULK1 initiation complex leads to activation of the class III phosphoinositide 3‐kinase (PI3K) complex. The PI3K complex, which is essential for vesicle nucleation, is composed of vacuolar protein sorting 34 (VPS34), ATG14, the activating molecule in BECN1‐regulated autophagy protein 1 (AMBRA1), and the scaffold protein Beclin‐1.^34^ Interaction of the complexes in steps 1 and 2 help to recluse proteins and lipids necessary for the AV formation.
3. This step is characterized by two ubiquitin‐like systems, the first system involves the formation of the ATG5‐ATG12‐ATG16 complex which is conjugated by the ATG7 and ATG10. In the second system, the protease ATG4 converts microtubule‐associated protein 1‐light chain 3 (LC3) into LC3‐I and stimulates the binding of ATG7 to attract ATG3 resulting in the ligation of phosphatidylethanolamine (PE) with LC3‐I to form LC3‐PE complex (also known as LC3‐II).^35^ In this process, a multitude of ATG5‐ATG12‐ATG16 and LC3‐II complexes are translocated from the cytoplasm to the autophagosome membrane. The ATG5‐ATG12‐ATG16 complex facilitates the protein lipidation of LC3 while LC3‐II supports membrane expansion as a scaffold protein in the formation of AVs.^36^
4. LC3‐II further acts in cargo recognition by directly interacting with cargo receptors such as sequestosome 1 (SQSTM1/p62) and neighbor of BRCA1 (NBR1).^37^ Cargo receptors are closely associated with the selective degradation of autophagy as specific cargo bind preferentially to a specific cargo receptor.^38^
5. ATG9 recruits lipid membrane which is derived from mitochondria, plasma membrane, Golgi, or the endoplasmic reticulum to the forming AVs to close the vesicle.^39^, ^40^, ^41^ When the isolation membrane is enclosed with trapped cargo, it is called the AV.
6. In this step, the AVs fuse with lysosomes to generate autophagolysosomes. Rab GTPases, membrane‐tethering complexes (HOPS complex, VPS genes), and soluble N‐ethylmaleimide‐sensitive factor attachment protein receptors (SNARE) involve in regulating this process.^42^
7. The membranes and the contents of the AV are degraded by lysosomal hydrolases. The autolysosome is broken down and degradation products, such as amino acids, peptides, and free fatty acids, are released into the cytoplasm to be reused, excreted into the bloodstream. These final products can be used for protein synthesis or can be used as source of energy for cell survival.^43^, ^44^

### 2.3 Autophagy‐related signaling pathways

**FIGURE 3:**
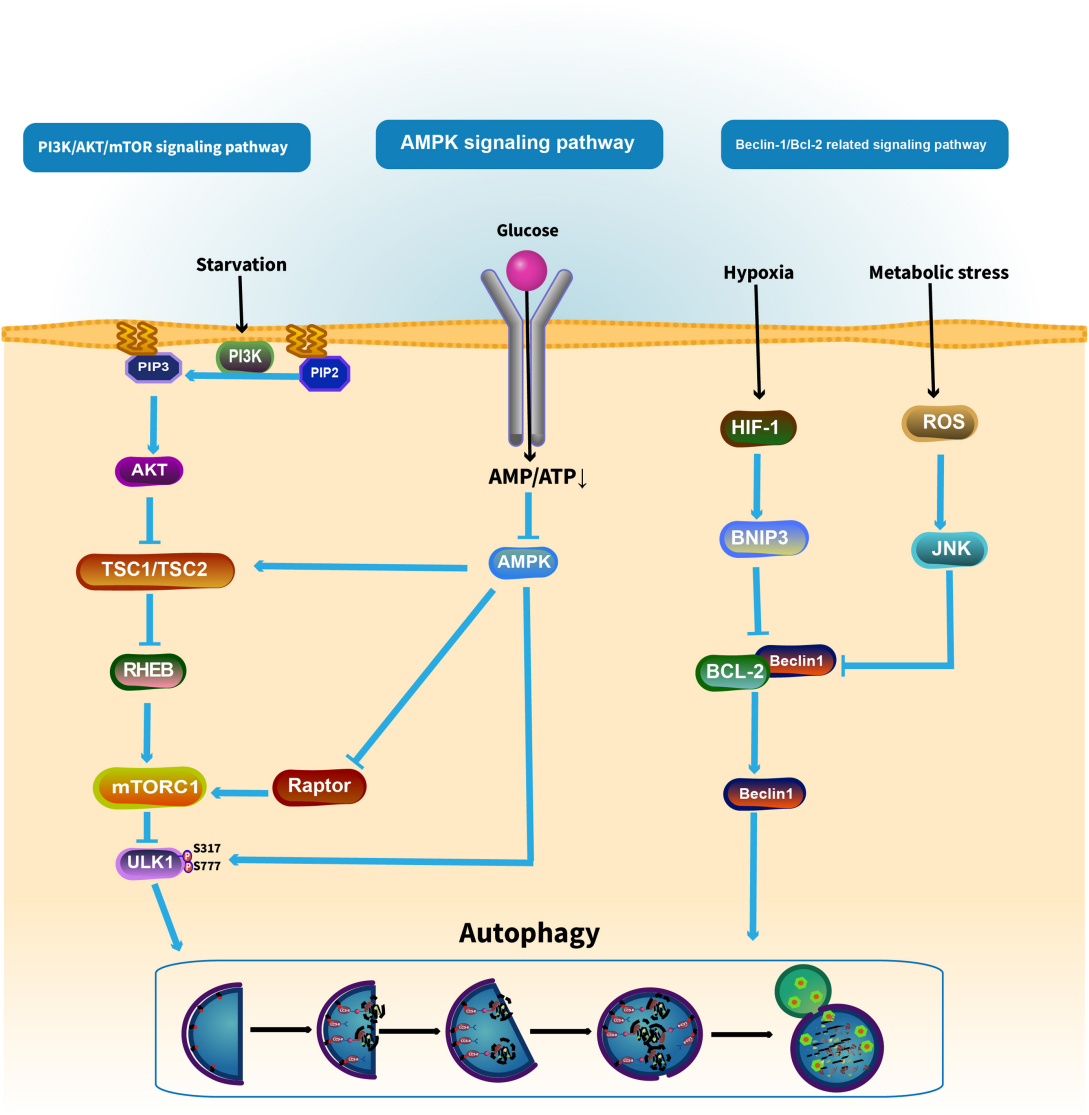
Autophagic pathways common in OS.

The regulation of autophagy is closely linked to distinct autophagy‐related signaling pathways, including (i) PI3K/protein kinase B (AKT)/mammalian target of rapamycin (mTOR) signaling pathway; (ii) adenosine monophosphate‐activated protein kinase (AMPK) signaling pathway; and (iii) Beclin‐1/Bcl‐2‐related signaling pathway (Figure 3).

#### 2.3.1 PI3K/AKT/mTOR signaling pathway

The class I PI3K (PIK3C1) is a class of lipid kinase whose main biochemical function is to catalyze phosphatidylinositol (PI) phosphorylation at the D3 position and convert phosphatidylinositol 4,5‐bisphosphate (PIP2) into phosphatidylinositol 3,4,5‐triphosphate (PIP3).^45^ PIP3 promotes AKT phosphorylation, and phosphorylated AKT directly catalyzes mTOR, ultimately leading to the inhibition of autophagy. On the other hand, PI3K signaling promotes AKT‐mediated phosphorylation and inactivation of the tuberous sclerosis complex 2 (TSC2) to disrupt the formation of the TSC1/TSC2 heterodimer, resulting in the production of RHEB, which then phosphorylates mTOR, causing the inhibition of autophagy.^46^ Under normal nutrient conditions, PI3K/AKT/mTOR signaling pathway blocks autophagy through inhibitory phosphorylation of ULK1 and the ATG14 subunit of VPS34 complexes.^47^ However, during periods of nutrient deprivation, PI3K/AKT/mTOR signaling pathway is rapidly inhibited and the repressive phosphorylation on ULK1 or VPS34 complexes is removed, thereby activating autophagy.

#### 2.3.2 AMPK signaling pathway

Another crucial signaling pathway AMPK, which inhibits autophagy through a variety of actions, including its regulatory effect on the MAPK/ERK pathway, the suppression of PI3K‐AKT/mTOR pathway, and the phosphorylation of ULK1 complex in autophagy.^48^ AMPK is a serine/threonine kinase, whose activity is sensitive to intracellular ratios of AMP/ADP to ATP. Under metabolic stress conditions, such as nutrient deprivation and energy depletion, a drop in ATP:AMP ratio results in activation of AMPK1, which can inhibit mTORC1 activity by activating the TSC1/TSC2 protein heterodimer, consequently inducing autophagy.^49^ AMPK also directly phosphorylates Raptor (a protein within the mTORC1), which leads to the inhibition of mTORC1.^50^ In addition, AMPK can activate ULK1 through direct phosphorylation of Ser317 and Ser777, or VPS34 lipid kinase through phosphorylation of the Beclin‐1 subunit to promote autophagy.^51^ Collectively, AMPK is an important autophagy activator, which is involved in the activation of autophagy through the abovementioned complex mechanisms.

#### 2.3.3 Beclin‐1/Bcl‐2‐related signaling pathway

Another important mechanism found to participate in autophagy regulation and tumorigenesis involves Beclin‐1, originally recognized as a Bcl‐2‐binding protein. It is a part of the PI3K complex, which initiates the formation of the phagophore. Beclin‐1 contains a BH3‐binding domain and has been found bound to Bcl‐2 in the form of a dual protein complex.^52^, ^53^ Importantly, the interaction of Bcl‐2 with the Beclin‐1 modulates PI3K complex and inactivates autophagy.^18^ Under normal nutrient conditions, Beclin‐1 is bound to Bcl‐2 inhibiting autophagy. During starvation or stressful conditions, Beclin‐1 is disrupted from Bcl‐2 through phosphorylation of the binding domain.^54^ In addition, c‐Jun N‐terminal kinase (JNK) is an important member of the MAPK protein family. Activated JNK can inhibit BCL2 combined with Beclin‐1, thus promoting the expression of Beclin‐1 and autophagy activation.^55^

## 3 THE ROLE OF AUTOPHAGY IN OS

### 3.1 Autophagy and OS proliferation

A growing number of studies have shown that autophagy positively regulates tumor cell proliferation in multiple cancer types. Cell proliferation is normally inhibited through downregulating autophagy‐related genes such as ATG7 in cervical cancer, breast cancer, and osteosarcoma.^56^, ^57^ Recently, Wang et al. indicated that microRNA‐22 could inhibit proliferation of OS cells through metadherin‐mediated autophagy.^58^ Metadherin, commonly known as astrocyte‐elevated gene‐1 (AEG‐1), is closely related to cancer initiation and progression.^59^ The expression of ATG5, phosphorylation of AMPK, and consequent induction of autophagy are all induced by AEG‐1.^60^, ^61^ However, the mechanism by which autophagy promotes proliferation of OS cells remains unclear. It has been reported that autophagy and cyclin can regulate each other.^62^ To demonstrate whether autophagy could regulate cell proliferation via modulating the levels of cyclins, OS cells were treated with autophagy inhibitors bafilomycin A1 or knocked down its ATG7. The results indicated that degradation of cyclin A2, which interacted with LC3B and p62/SQSTM1, was suppressed in G2/M phase but not in G1/S phase. Besides, ATG7 knockdown prolonged mitotic time.^57^ Thus, we can infer that autophagy promotes tumor cell proliferation through degrading cyclin A2, thereby reducing G2/M cell cycle arrest. Whether other cyclins are involved in autophagy‐promoting proliferation of OS cells requires more further studies.

In the previous paragraph, we explored that autophagy could positively regulate OS cell proliferation. However, a mass of studies has pointed out that activation of autophagy could inhibit proliferation of OS cells.

BECN1 was the first tumor suppressor to link autophagy to tumors, and its monoallelic deletions have been found in as many as 40% to 75% of various human tumors, such as prostate, breast, and ovaries.^15^, ^63^ It has been reported that low expression of BECN1 suppresses autophagy and positively regulates cell proliferation.^64^, ^65^ Recently, Parlayan et al. explored the role of BECN1 in proliferation of OS cells. Their findings suggest that AT‐rich interacting domain 3A (ARID3A) could suppress proliferation of OS cells through regulating BECN1 expression.^66^ What’ s more, rapamycin could suppress proliferation of MG‐63 cells through upregulating Beclin‐1 (encoded by BECN1) protein level and activating autophagy.^67^ These studies suggest that BECN1 could function as a potential OS therapeutic target which allows by activating autophagy.

### 3.2 Autophagy and OS metastasis

Some important regulatory pathways such as PI3K/AKT/mTORC1 pathway and ERK/MAPK pathway similarly play an important role in activating autophagy and inhibiting proliferation of OS cells. Liu et al found that ginsenoside‐Rg5 (Rg5) reduced the phosphorylation of PI3K, Akt, and mTORC1 activation. Conversely, LC3‐mediated autophagy upregulated significantly. More importantly, Rg5 inhibited proliferation of OS cells potently in a dose‐dependent manner.^68^ Sprouty‐related EVH1 domain protein 2 (SPRED2) is a repressor of ERK/MAPK pathway which plays an important role in activating autophagy. A recent study indicated that miR‐19 promoted cell proliferation via suppressing SPRED2‐mediated autophagy in OS cells.^69^ Overall, these studies demonstrate that autophagy‐related genes and pathways may serve as potential targets for inhibiting proliferation of OS cells. Meanwhile, we take the view that more detailed studies are needed to distinguish whether autophagy positively regulates or negatively regulates proliferation of OS cells, so as to facilitate the development of precise targeted therapeutic drugs in the future. A summary of the relationship between autophagy and OS proliferation is listed in Table S1.

### 3.3 Autophagy and OS chemotherapy

The 5‐year survival rate in patients with nonmetastatic OS could reach 75%; however, this value is just 20% in metastatic patients.^70^, ^71^ The OS cells exhibit a high propensity to proliferate and are prone to metastases, which appears to be the most critical internal reason for inferior prognosis of OS patients.^72^ Recently, more and more researches have shown that autophagy plays an important role in metastasis of OS. Zhang et al. pointed out that COP9 signalosome subunit 3 (COPS3) knockdown decreased OS metastasis through suppressing Beclin‐1.^73^ Similarly, Long et al. showed that valosin‐containing protein (VCP) overexpression activated the ERK/NF‐κβ/Beclin‐1 pathway, enhanced autophagy‐mediated anoikis resistance, and promoted metastasis in OS.^74^ Furthermore, a recent study found that programmed death ligand‐2 (PD‐L2) knockdown‐induced attenuation of autophagy inhibited metastasis and invasion of OS cells which occurred through RhoA‐ROCK‐LIMK2 signaling pathway. During this process, when PD‐L2 was knocked down in OS cells, reduced levels of LC3‐II and Beclin‐1 were observed through western blotting.^75^ Previous studies have pointed out that autophagy could be regulated through the mTOR signal transduction pathway.^76^ The mTOR signal transduction pathway is also critical for the regulation of OS metastasis. Zhao et al. are the first to demonstrate that tumor‐suppressing STF cDNA 3 (TSSC3) induces autophagy through suppressing the Src‐dependent PI3K/Akt/mTOR pathway in OS. Besides, they pointed out that TSSC3‐induced autophagy contributed to inhibiting metastasis of OS, both in vitro and in vivo.^77^ Results from another team showed that knockdown of Aurora‐B stimulated autophagy through reducing mTOR/ULK1 and resulted in suppressing OS metastasis.^78^ In summary, Beclin‐1 and mTOR‐related signaling pathways are expected to be therapeutic targets for inhibiting OS metastasis which will become the focus of future researches. A summary of the relationship between autophagy and OS metastasis is listed in Table S1.

Neoadjuvant chemotherapy regimen consisting of MTX, DOX, and CIS were first proposed in the 1970s as a means to shrink primary lesions, minimize surgical complications, and treat distant micrometastases, significantly improving overall survival in OS patients. Although neoadjuvant chemotherapy has significantly improved survival rate in OS patients, a mass of patients subsequently develops chemoresistance, which is now the major cause of higher mortality.^79^ A large number of studies have pointed out that multiple chemotherapeutic drugs could induce autophagy.^80^, ^81^ In addition, tumor cells frequently utilize the autophagy pathway to promote chemoresistance and improve survival ability.^82^

Recently, some researches have shown that chemotherapy‐resistant OS cells are often accompanied by an increased autophagy.^83^, ^84^, ^85^ Hence, increasing the chemosensitivity of OS through suppressing autophagy has become the focus of study. Tang et al. pointed out that Sestrin2 promoted chemoresistance in OS cells by increasing autophagy. Further mechanism experiments suggested that Sestrin2 activated autophagy through suppressing mTOR via the PERK‐eIF2α‐CHOP pathway.^86^ Similarly, HSP90AA1, is a key factor in promoting the development of OS chemoresistance in vitro and in vivo, could induce autophagy via PI3K/Akt/mTOR pathway.^87^ It was revealed by another study that wnt/β‐catenin signaling pathway activation reversed gemcitabine resistance through inhibiting Beclin‐1‐mediated autophagy in MG63 OS cell.^88^ In recent years, miRNAs have emerged as key factors regulating OS chemosensitivity or chemoresistance through targeting autophagy‐related genes or pathways. Most miRNAs increase chemosensitivity of OS through inhibiting autophagy. Some studies pointed out that miR‐22 could decrease CIS resistance through suppressing metadherin (MTDH)‐mediated autophagy in MG‐63 cells.^89^ Consistent with these findings, miRNA‐22 could also regulate the CIS resistance of OS cells by inhibiting autophagy through PI3K/Akt/mTOR pathway.^90^ Gao et al. demonstrated that miR‐375 suppressed autophagy through targeting autophagy‐related 2B (ATG2B) in CIS‐resistant OS cells.^91^ Furthermore, a novel axis of SNHG16/miR‐16/ATG4B promoted OS chemoresistance through inducing autophagy.^92^ Acting as “sponges” for complementary miRNA, several lncRNAs have been shown to play a critical role in OS chemoresistance. The lncRNA CTA reversed DOX resistance by targeting miR‐210 and negatively regulating autophagy.^93^ LncRNA Sox2OT‐V7 promoted OS cell autophagy and induced DOX chemoresistance through targeting miR‐142/miR‐22.^94^ The association between autophagy and chemoresistance in OS is rather complicated and much of the mechanism has not been elucidated. However, from the analysis of existing research, targeting miRNAs provides new targets for the development of novel antitumor drugs.

Although numerous studies demonstrated that chemotherapy triggered a cytoprotective autophagy, autophagic cell death has received increasing attention. Autophagic cell death is a type of cell death mediated by autophagy which is distinct from apoptosis or necrosis.^95^ It differs from cytoprotective autophagy and could be increased through autophagy activation or decreased through autophagy inhibition. There was a report which showed that flavonoid luteolin could upregulate beclin‐1 to enhance DOX‐induced autophagy, which caused U2OS cell death.^96^ Similarly, as an autophagy inducer, voacamine could enhance the chemosensitivity of DOX‐resistant U2OS cells through activating autophagic cell death.^97^ More importantly, a recent study of Liao et al. pointed out that CXCR4 blocker increased DOX sensitivity of OS through activating autophagic cell death by negatively regulating PI3K/AKT/mTOR signaling pathway.^98^

We conclude from above that mild autophagy could increase chemoresistance because of cytoprotective effect, whereas excessive autophagy reverses chemoresistance through activating cell death (Table 1). Thus, our study has important guiding significance in developing autophagy inhibitors or autophagy activators to modulate chemoresistance of OS in the future.

**TABLE 1:** Relationship between autophagy and chemoresistance in OS

First author, year	OS cell types	Target gene/signaling pathway	Autophagy‐related molecules	Autophagy activation/inhibition	Chemotherapeutic drugs	Chemoresistance
Tang, 2021^86^	MG‐63, HOS, 143B	PERK‐eIF2α‐CHOP, mTOR	Sestrin2	activation	MTX, DOX, CIS	up
Xiao, 2018^87^	MG‐63, Saos‐2, U‐2	PI3K/Akt/mTOR	HSP90AA1	activation	MTX, DOX, CIS	up
Tao, 2017^88^	MG‐63	wnt/β‐catenin	Beclin‐1	inhibition	GEM	down
Meng, 2020^89^	MG‐63	miR‐22	MTDH	inhibition	CIS	down
Meng, 2020^90^	MG‐63, U‐2, Saos‐2, OS9901	miRNA‐22, PI3K/Akt/mTOR	LC3, ATG5	inhibition	CIS	down
Gao, 2020^91^	MG‐63, U‐2	miR‐375	ATG2B	inhibition	CIS	down
Liu, 2019^92^	SAOS2, U‐2	SNHG16, miR‐16	ATG4B	activation	CIS	up
Wang, 2017^93^	Saos‐2, U‐2, MG‐63	lncRNA CTA, miR‐210	LC3B	inhibition	DOX	down
Zhu, 2020^94^	MG‐63, U‐2, Saos‐2, HOS	LncRNA Sox2OT‐V7, miR‐142/miR‐22	LC3II, Beclin‐1	activation	DOX	up
Miao, 2015^96^	MG‐63	PI3K	Beclin‐1	activation	DOX	down
Meschini, 2007^97^	U‐2	‐	‐	activation	DOX	down
Liao, 2021^98^	LM8, Dunn	CXCR4, PI3K/AKT/mTOR	Beclin‐1, LC3B	activation	DOX	down

### 3.4 Autophagy and OS radiotherapy

Local radiotherapy has been found to have some positive effects in OS patients who are inoperable and poorly responsive to chemotherapy.^99^ Due to the radiotherapy insensitivity of OS, the exploration of clinically effective radiosensitizers has attracted more and more attention of researchers. Hypoxia‐induced autophagy has been reported to enhance OS cell survival through enhancing radioresistance. Their further studies indicated that hypoxia‐mediated expression of HIF‐1α increased LC3 levels and accelerated the scavenging of cellular ROS products.^100^ Additionally, Jin et al. found that inhibition of HIF‐1α effectively decreased hypoxia‐induced transcription and enhanced radiosensitivity of hypoxic MG‐63 OS cells.^101^ At the same time, Ding et al. confirmed that the molecule 3MA also increased the radiosensitivity of MG63 cells through negatively regulating autophagy.^102^ Oh et al. further clarified the association between autophagy and radioresistance through utilizing highly linear energy transfer (LET) neutron radiation. Their study indicated that high LET neutron radiation increased autophagy through effectively suppressing the Akt–mTOR pathway and negatively regulating p‐mTOR expression.^103^

It has been reported that activation of the nuclear factor epithelioid 2‐related factor 2 (NRF2) pathway conferred resistance to radiation therapy in U‐2 OS cell. And this process was regulated by autophagy‐mediated activation of ERK 1/2 kinases.^104^ Taken together, these findings collectively suggest that targeting autophagy may be an important means to improve OS radiosensitivity. A summary of the relationship between autophagy and OS radiotherapy is listed in Table S1.

### 3.5 Autophagy and OS immunotherapy

Over the past few years, there has been increasing evidence that the immune system plays a critical role in progression and treatment of tumors. The most studied is the improvement of cancer immunotherapy by blocking PD‐1/PD‐L1 immune checkpoints. In recent years, enhancing the efficacy of antitumor immunotherapy through autophagy has become a promising therapeutic approach. Tumor‐promoting effects of autophagy could be abrogated through immune checkpoint blockers (ICBs) in combination with autophagy inhibitors.^105^ Young et al. demonstrated that synergistic blockade of tumor cell autophagy improved the efficacy of PD‐1/PD‐L1 blockers in colon, breast, and pancreatic cancer models.^105^, ^106^ In another study, Huntington‐interacting protein 1‐related (HIP1R), an autophagy receptor for PD‐L1 binding, induced PD‐L1 degradation in lysosomes and subsequently suppressed tumor growth by activating T cells.^107^ Therefore, autophagy‐targeted modulators might be used as potential sensitizers to enhance the effective clinical response of the tumor to ICBs.

Consistently, encouraging results are obtained from immunotherapy in advanced, metastatic, and recurrent OS.^108^, ^109^ Yu et al.^110^ pointed out that combination therapy of PDT with the autophagy inhibitor 3‐MA enhanced immune cytotoxicity through negatively regulating the PD‐L1 immune checkpoint. Further in vivo and in vitro experiments found that CD8^+^ T cells were activated to inhibit OS growth and metastasis. Meanwhile, Ren et al.^75^ found that PD‐L2 blockade reduced OS migration and invasion rates through antagonizing RhoA‐ROCK‐LIMK2 signaling, inhibiting epithelial–mesenchymal transition (EMT), and suppressing autophagy by negatively regulating Beclin‐1 expression. Additionally, CBZP, a pH‐sensitive autophagy‐controlling nanocarrier, could enhance the tumor immunogenicity and sensitize the antitumor T‐cell immunity through inducing autophagic cell death.^111^ Altogether, we take the view that targeting the autophagy could enhance and/or synergize OS immunotherapy. A summary of the relationship between autophagy and OS immunotherapy is listed in Table S1.

## 4 CONCLUSION

In this review, we summarized the role of autophagy in OS proliferation, metastasis, chemotherapy, radiotherapy, and immunotherapy. Overall, autophagy plays a dual role in different stages of OS. However, there is no specific mechanism that defines this dual role of autophagy in OS. Until now, clinical outcomes of OS have not met expectations. A growing number of studies have confirmed that autophagy plays a dual role in regulating OS chemoresistance through exerting cytoprotective effects or causing autophagic cell death. Hence, either inhibition of cytoprotective autophagy or enhancement of autophagic cell death can improve chemosensitivity of OS. Besides, autophagy inhibition can enhance the efficacy of radiotherapy and immunotherapy in OS. Although autophagy regulator has great potential in the treatment of OS, the practical utility of all kinds of autophagy regulators in clinical practice remains to be investigated. We look forward to more specific autophagy modulators which could improve the efficacy of anti‐OS therapies will be found in the near years.

## AUTHOR CONTRIBUTIONS

**Biao Ning:** Conceptualization (equal); data curation (equal); formal analysis (equal); project administration (equal); resources (equal); visualization (equal); writing – original draft (lead); writing – review and editing (lead). **Yixin Liu:** Data curation (equal); formal analysis (equal); investigation (equal); resources (equal). **Tianhe Huang:** Formal analysis (equal); methodology (equal); project administration (equal); supervision (equal); validation (equal). **Yongchang Wei:** Conceptualization (lead); funding acquisition (lead); methodology (lead); project administration (lead); resources (lead); supervision (lead).

## FUNDING INFORMATION

There is no funding for this review.

## CONFLICT OF INTEREST STATEMENT

All authors acknowledge that there is no conflict of interest in this review.

## ETHICAL APPROVAL

This is a review article and the need for ethics approval and consent was waived.

## Supporting information


Table S1.

## Data Availability

This is a review article and therefore the data utilized in this review are open access.
